# Outcome of Teriparatide Treatment on Fracture Healing Complications and Symptomatic Bone Marrow Edema in Four Adult Patients With Hypophosphatasia

**DOI:** 10.1002/jbm4.10215

**Published:** 2019-08-28

**Authors:** Tobias Schmidt, Tim Rolvien, Carolin Linke, Nico Maximilian Jandl, Ralf Oheim, Michael Amling, Florian Barvencik

**Affiliations:** ^1^ Department of Osteology and Biomechanics University Medical Center Hamburg‐Eppendorf Hamburg Germany; ^2^ Department of Orthopedics University Medical Center Hamburg‐Eppendorf Hamburg Germany

**Keywords:** HYPOPHOSPHATASIA, ALPL, TERIPARATIDE, PYRIDOXAL‐5‐PHOSPHATE

## Abstract

The response to teriparatide has been described in very few cases of hypophosphatasia (HPP). In this cross‐sectional study, we report the prevalence of symptomatic bone marrow edema (BME) and fracture healing complications in a large cohort of childhood and adult HPP patients and discuss the results of teriparatide treatment in four cases. From 2016 to 2018, 51 patients with a diagnosis of HPP were seen at our institution. The diagnosis of HPP was established by low serum alkaline phosphatase (ALP), elevated serum pyridoxal‐5‐phosphate (PLP), at least one typical clinical symptom of HPP and supported by ALPL mutation analysis. In this study cohort, 28 (56%) and 14 (27%) patients had a history of fracture or a history of BME, respectively. Four patients, including middle‐aged to elderly women and men who all presented with persistent symptomatic BME or fracture healing complications, were treated with teriparatide. DXA was performed prior to treatment and laboratory values were measured on a regular basis during treatment. Treatment with teriparatide showed variable effects in terms of clinical and biochemical response. Although all four patients displayed a temporary increase in ALP activity, only two patients with a mild form of adult HPP and moderately increased PLP levels showed definite clinical and radiological improvement after teriparatide treatment. In conclusion, fracture healing complications and BME occur frequently in HPP patients. Teriparatide shows variable clinical and biochemical effects depending on the severity of the disease. PLP levels and the number of ALPL alleles might be good parameters to predict treatment outcomes. © 2019 The Authors. *JBMR Plus* Published by Wiley Periodicals, Inc. on behalf of the American Society for Bone and Mineral Research

## Introduction

Hypophosphatasia (HPP) is a rare inherited disease caused by a loss of function mutation within the gene ALPL encoding the tissue nonspecific alkaline phosphatase (TNSALP).^1^ Deficient alkaline phosphatase (ALP) activity leads to an accumulation of its substrates including pyridoxal‐5‐phosphate (PLP) and inorganic pyrophosphate (PPi). The increase of PPi causes impaired skeletal mineralization by blocking hydroxyapatite crystal formation, thus predisposing an individual to fractures, fracture healing complications, and bone marrow edema (BME).^1^, ^2^

HPP is currently classified into six forms based on the age of onset of clinical manifestation and the severity of the disease: perinatal, benign perinatal, infantile, childhood, adult, and odontohypophosphatasia.^3^ Although the perinatal and infantile forms are associated with severe reduction of mineralized bone and high mortality caused by respiratory failure, the adult form shows a highly variable clinical severity.^1^ These patients suffer from fractures, osteomalacia, muscle pain, recurring headaches, and intra‐articular calcium PPi dehydrate crystal deposition (CPPD).^4^, ^5^ The severity of the clinical manifestation correlates with the accumulation of ALP substrates, in particular PLP.^5^, ^6^ Patients suffering from the adult form frequently present with recurrent stress fractures of the metatarsals and subtrochanteric or diaphyseal femoral fractures.^4^, ^5^, ^7^, ^8^ Most of these fractures are associated with slow and often delayed fracture healing and can progress to more complicated fractures. The therapy options for these fracture healing complications are still very limited.

BME has been reported in the juvenile form of HPP,^9^ but not in the adult form of HPP. Interestingly, studies in pediatric patients suggest that recurrent BME in HPP can mimic chronic multifocal nonbacterial osteomyelitis (CRMO) without fulfilling all criteria for the diagnosis.^9^, ^10^ Although CRMO has been reported in HPP, it is not clear if the frequently occurring bone pain in HPP is associated with recurring BME. Similarly, there are no established therapies for persistent BME in HPP patients. Therefore, we report the effects of the osteoanabolic drug teriparatide on BME in 2 HPP patients.

The approval of asfotase alfa (AA; Strensiq; Alexion Pharmaceuticals, Boston, MA, USA) in 2015 has strongly improved the treatment options for HPP patients.^11^ AA is a bone‐targeted enzyme replacement therapy that has been shown to improve skeletal health, growth, muscle strength, and pain level in infantile and childhood HPP. Currently, AA is only approved for pediatric‐onset HPP worldwide, with the exception of Japan, where AA is approved for all ages. Hence, there is yet no approved medical therapy for adult‐onset HPP patients in Europe and North America, who had no symptoms in childhood or adolescence. Off‐label use of teriparatide has first been reported in adults in 2007,^12^ and showed variable clinical effects in further studies.^12^, ^13^, ^14^, ^15^, ^16^, ^17^ In 2017, the treatment of 8 adult HPP patients with a monoclonal antisclerostin antibody (BPS804) increased lumbar BMD and bone formation markers,^18^ but no data on fracture healing or BME were presented.

In this study, we report the frequency of bone fractures, fracture healing complications, and BME in a large cohort of childhood‐ and adult‐onset HPP. We report four cases that were treated with teriparatide for symptomatic BME or a fracture healing complication that showed variable clinical and biochemical effects.

## Patients and Methods

### Study group

Between 2016 and 2018, 51 HPP patients with a complete medical record, imaging diagnostics, and positive genetic findings were seen at the Department of Osteology and Biomechanics, at the University Medical Center Hamburg‐Eppendorf, Germany. No patient with a diagnosis of HPP who was seen during that time was excluded from the study. The mean age and BMI was 46.0 ± 18.8 years and 25.38 ± 4.6 kg/m^2^, respectively. Of the 51 patients, 39 (76.4%) were women and 12 were men (23.6%). Six (12%) patients were younger than 18 years of age; 29 patients (56.9%) had a reported history of fracture; 12 patients (23.5%) had suffered from a fracture within the last 24 months of presentation. Fourteen patients (27.4%) had a history of symptomatic BME that had been diagnosed using MRI. Five of these patients (9.8%) suffered from BME at the time of presentation at our institution with 3 patients suffering from persistent BME (>6 months). Nine patients (17.6%) had a history of both a fracture and a BME. All patients in this study were white. A diagnosis of HPP was established by the combination of persistent low serum ALP, raised PLP levels, and typical clinical symptoms (early loss of deciduous or permanent teeth, rickets, fractures, CPPD, delayed fracture healing). All patients had at least one typical HPP symptom. In all patients, the diagnosis of HPP was supported by genetic testing. In all patients a medical history of fractures, bone marrow edema, and fracture healing complication were available. All patients were explicitly asked for a history of fracture or a BME. However, the MRI images of BME in 2 patients and the radiographs of 12 patients with a history of fractures were not available. The characteristics of all patients with a BME are summarized in Supplemental Table 1. Four adult patients received teriparatide (20 μg by daily s.c. injection) for different periods. The length of therapy was determined on the basis of the clinical outcome of the fracture healing complication or the BME. Patients were taking teriparatide as recommended on a daily basis. Another patient with a fracture healing complication was recommended for the off‐label use of teriparatide, but did not follow the recommendation. Informed written consent for publication was taken from all patients of the study cohort. The local ethics committee approved the retrospective study (WF‐77/18).

**Table 1:** Demographic, Clinical, and Laboratory Data of All Hypophosphatasia Patients

		Mean ± SD/*n* (%)	Mean ± SD/ *n* (%)
Sex (f/m)		39/12	
Age (years)		46.0	
0–10		0/0 (0)	
11–17		6/51 (9.8)	
18–30		7/51 (15.7)	
31–49		14/51 (27.5)	
>50		24/51 (47.1)	
Clinical forms			
Infantile		8/51 (15.7)	
Childhood		12/51 (23.5)	
Adult		31/51 (60.8)	
Measuring units		<18 years (*n* = 6)	>18 years (*n* = 45)
Weight (kg)		59.8 ± 12.3	72.8 ± 14.1
Weight (pounds)		131.9 ± 27.1	160.5 ± 30.98
Height (m)		1.68 ± 0.11	1.67 ± 0.1
Height (inches)		66.34 ± 4.15	66.11 ± 3.6
Height (feet)		5.53 ± 0.35	5.5 ± 0.3
BMI (kg/m^2^)		21.1 ± 4.14	25.9 ± 4.4
Laboratory data for patients >18 years (*n* = 46)	Reference values for adults	<18 years (*n* = 6)	>18 years (*n* = 45)
Calcium (mmol/L)	2.13–2.63	2.25 ± 0.6	2.30 ± 0.1
Phosphate (mmol/L)	0.77–1.50	1.33 ± 0.25	1.13 ± 0.26
Parathyroid hormone (ng/L)	17–84	53.4 ± 9.7	53.4 ± 35.5
Osteocalcin (µg/L)	5.4–59.1	50.7 ± 22.6	15.04 ± 5.5
Pyridoxal‐5‐phosphate (µg/L)	<18.5	44.7 ± 21.9	93.36 ± 149.2
Alkaline phosphatase (U/L)	35–104	73.00 ± 22.7	24.7 ± 7.9
Bone specific alkaline phosphatase (µg/L)	5.2–24.4	19.4 ± 6.6	3.9 ± 1.6
25‐OH‐D (μg/L)	>30	21.7 ± 10.6	29.7 ± 12.2
Vitamin D deficiency	<20 μg/L	2/6 (33.3%)	10/45 (22.2%)
Vitamin D insuffficiency	<30 μg/L	2/6 (33.3%)	17/45 (37.8%)
Clinical history		<18 years (*n* = 6)	>18 years (*n* = 45)
Bone marrow edema		1/6 (16.7%)	13/45 (28.9%)
Fracture		2/6 (33.3%)	27/45 (60.0%)

### Dual‐energy X‐ray absorptiometry

BMD was measured by DXA (Lunar iDXA; GE Healthcare; Madison, WI, USA). Two skeletal areas, the left proximal femur and the lumbar spine (L1 to L4), were evaluated by DXA. The patients were placed in the supine position and scanned according to the manufacturer's instructions. The detected BMD of the projected bone area was expressed in grams per square centimeter (g/cm^2^), and the corresponding *T*‐ and *Z*‐scores were generated by the software supplied by the manufacturer.

### Laboratory test

Biochemical analyses of bone metabolism markers, including serum levels of pyridoxal‐5‐phosphate, AP, bone‐specific alkaline phosphatase (BAP), 25(OH)D3, calcium, osteocalcin, phosphate, PTH, and osteocalcin were performed by the Department of Clinical Chemistry, University Medical Centre Hamburg‐Eppendorf (Germany). No patients were taking vitamin B6; however, patients taking multivitamin tablets were instructed to pause for 14 days before blood collection. The blood was taken with a random clinical venipuncture. Vitamin D insufficiency was defined as a 25‐OH‐D level <30 μg/L and deficiency as levels <20 μg/L.

### Bone biopsy

After informed consent, an iliac crest biopsy was performed in 1 patient (case 4) because of chronic kidney disease (grade 3) according to the KDIGO guideline for chronic kidney disease–mineral and bone disorder (CKD‐MBD) (https://kdigo.org/guidelines/ckd‐mbd/). Tetracycline labeling was to be performed; however, it was stopped after the first dose because of allergic symptoms of the patient. The bone biopsy was dissected 2 cm below and 2 cm behind the crista iliac superior anterior according to Bordier,^19^ and fixed overnight at 4°C in 4% PBS‐buffered formaldehyde. After dehydration in an ascending concentration of ethanol, the biopsy was embedded nondecalcified in methyl methacrylate and cut into 5‐μm thick sections. The sections were stained according to standard protocol after von Kossa/van Gieson as described.^20^, ^21^ Static bone histomorphometry parameters were evaluated according to the ASBMR standards^22^ using the Osteomeasure image analysis system (OsteoMetrics, Decatur, GA, USA). Bone mineral density distribution was analyzed by quantitative backscattered electron imaging and performed on nondecalcified methyl methacrylate‐embedded bone biopsy as described previously.^23^, ^24^ The scanning electron microscope (LEO 435VP; LEO Electron Microscopy Ltd., Cambridge, England) was operated at 20 kV and 665 pA at a constant working distance (BSE Detector, type 202; K.E. Developments Ltd., Cambridge, England).

## Results

**Figure 1:**
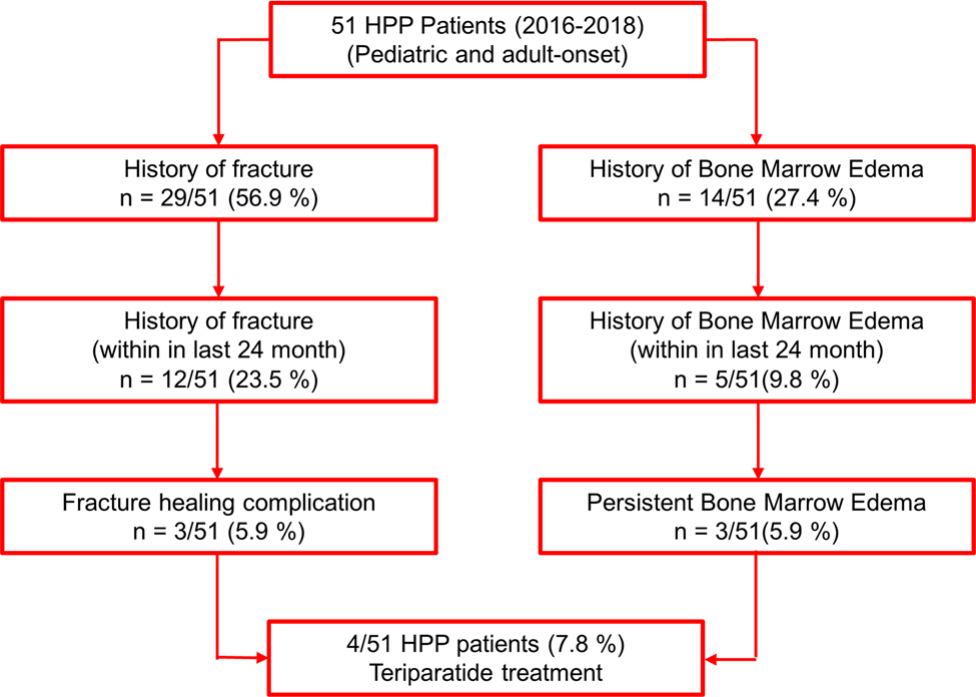
Flowchart of the hypophosphatasia patient's cohort with numbers of patients suffering from fractures and bone marrow edema.

Over 2 years, 51 HPP patients with a complete medical record, imaging diagnostics, and genetic findings were seen at our institution. The demographic, clinical, and laboratory data of the patients cohort are summarized in Table 1. Fracture occurrence, fracture healing complications, and BME in the study group are outlined in Fig. 1. The characteristics of all patients with a BME are summarized in Supplemental Table 1. Here, we did not identify any specific risk factors with regard to demographic or laboratory data (data are not shown).

**Figure 2:**
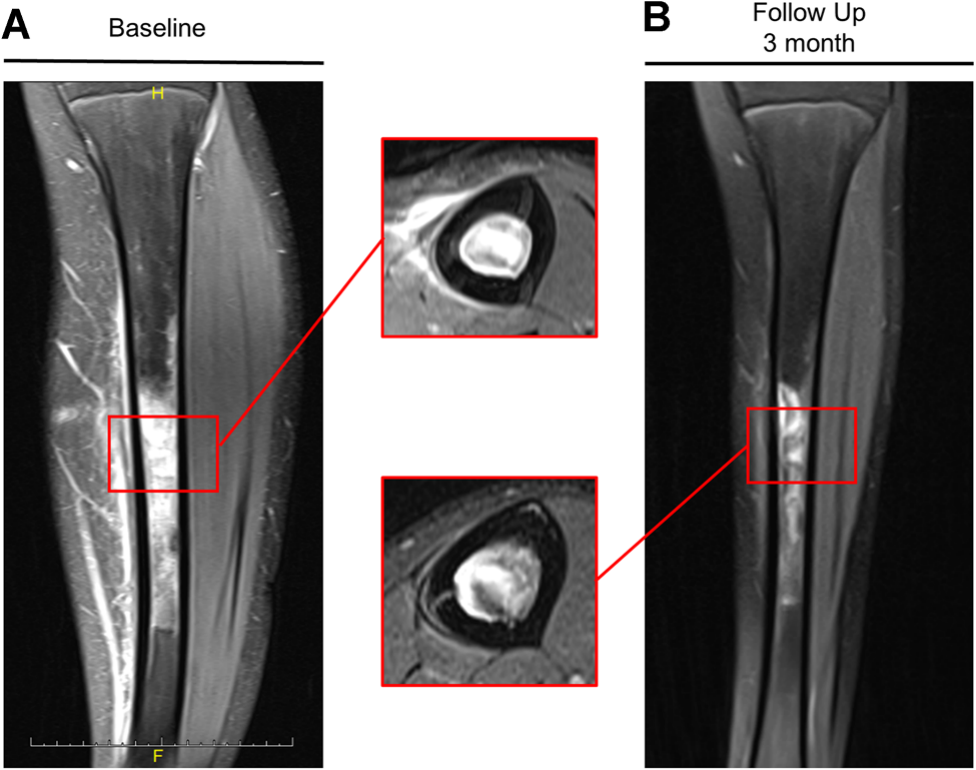
Coronal T2 MR images of a 14‐year‐old female patient at (*A*) initial presentation and (*B*) at follow‐up after 3 months demonstrating bone marrow edema of the tibia diaphysis. The patient was an active handball player and suffered from persistent pain at the tibia. She was first diagnosed with osteomyelitis and treated with antibiotics until transferred to our institution. Laboratory assessment revealed low alkaline phosphatase activity and elevated pyridoxal‐5‐phosphate levels. TNSALP mutation analysis showed a heterozygous mutation (c.746G>T) and the patient was diagnosed with hypophosphatasia.

An example of a persistent symptomatic BME is provided in Fig. 2. This 14‐year‐old girl was an active handball player for many years and suffered from pain in the left lower leg. The girl had experienced menarche at the age of 13 years. After 3 months of persistent pain, an MRI was performed showing BME of the tibia diaphysis (Fig. 2A). Subsequently, the patient was treated with antibiotics for 6 weeks until transferred to our institution for a second opinion. At the time of presentation, a broad laboratory workup was performed; it was completely unremarkable, including blood count and leukocyte differential indices. However, osteologic assessment revealed age‐ and gender‐adjusted low ALP activity with 58 U/L (age‐ and gender‐adjusted reference value of 78 to 120 U/L) and a corresponding elevated PLP level of 59.7 μg/L (reference value <18.5 μg/L). ALPL mutation analysis showed a heterozygous mutation (c.746G>T). The patient had no other clinical symptoms, had not shed deciduous teeth prematurely, and was otherwise healthy. Therefore, we did not treat the patient with AA. However, antibiotic therapy was stopped and the patient was treated with nonsteroidal anti‐inflammatory drugs, which greatly reduced the pain. An MRI was repeated after 3 months (Fig. 2B); it showed moderate resolution of BME.

Below we report four additional cases that presented with either BME or delayed fracture healing over a period of 2 years and were treated with teriparatide. All demographic, clinical, genetic, and laboratory data of these four clinical cases are summarized in Table 2.

**Table 2:** Demographic, Clinical, Genetic, DXA, and Laboratory Data of Hypophosphatasia Patients Who Had Been Treated With Teriparatide

		Case 1	Case 2	Case 3	Case 4
Sex (F/M)		F	M	F	M
Age		55	48	68	48
Weight (kg)		70.5	96.7	61.5	65.0
Height (m)		1.67	1.98	1.65	1.75
BMI (kg/m^2^)		24.6	24.5	22.6	21.22
DXA					
*T*‐score lumbar spine		–2.1	–1.2	–2.0	−2.4
*Z*‐score lumbar spine		–1.3	–1.3	–0.2	−1.8
*T*‐score femoral neck		–1.4	–1.2	–3.2	−2.4
*Z*‐score femoral neck		–0.6	–1.3	–1.9	−1.7
Laboratory data	Reference values				
Calcium (mmol/L)	2.13–2.63	2.29	2.36	2.20	2.10
Phosphate (mmol/L)	0.77–1.50	1.28	0.97	1.19	1.72
Parathyroid hormone (ng/L)	17–84	37.4	24.9	68.0	113.1
Osteocalcin (µg/L)	5.4–59.1	10.4	18.7	8.4	18.9
Pyridoxal‐5‐phosphate (µg/L)	<18.5	38.5	27.3	26.2	400
Alkaline phosphatase (U/L)	35–104	17	28	28	10
Bone specific alkaline phosphatase (µg/L)	5.2–24.4	1.8	6.9	3.3	1.5
25‐OH‐D (μg/L)	>30	29.6	30.4	40.5	17.2
ALPL mutation		c.1001G>A (p.Gly334Asp)	c.455G>A (p.R152H)	c.535G>A (p.A179T)	c.746G>T (p.Gly249Val) + c.625A>T (p.Met209Leuc)
		*Heterozygote*	*Heterozygote*	*Heterozygote*	*Compound heterozygote*

All values are prior to treatment.

## Case 1

**Figure 3:**
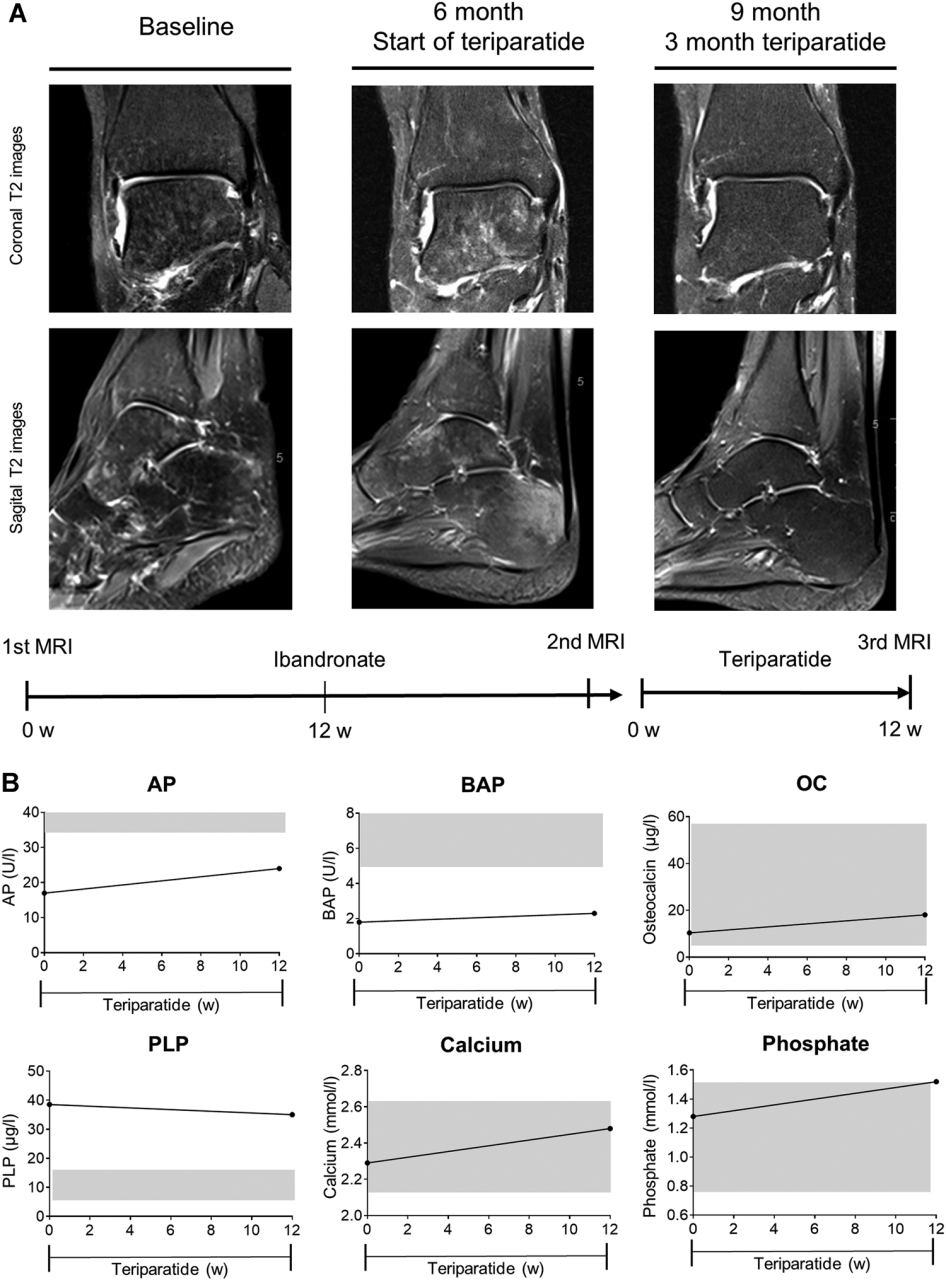
Case 1 (*A*) Coronal T2 (top) and sagittal T2 (bottom) MR images of the right foot at baseline, after 6 months of bisphosphonate therapy, and at follow‐up after 3 months of teriparatide therapy. (*B*) Biochemical response with levels of alkaline phosphatase (ALP), bone‐specific alkaline phosphatase (BAP), osteocalcin (OC), pyridoxal 5’‐phosphate (PLP), calcium, and phosphate after cessation of treatment with ibandronate and onset of therapy with teriparatide. Grey areas represent reference range. MRI showed improvement and the laboratory report displayed a positive biochemical response.

This 55‐year‐old woman had suffered from persistent pain after a right ankle sprain caused by a twisting trauma while walking. She was treated with an ankle–foot orthosis for 6 weeks with full load on the right foot; she carried out normal everyday activities after that. However, no sport was undertaken because of pain during exercise. After 3 months, an MRI was performed showing moderate diffuse bone edema of the talus and calcaneus (Fig. 3A). The patient was then treated twice with an i.v. bisphosphonate (ibandronate). After 6 months, a new MRI was performed before the patient presented at our institution. Here, the MRI showed worsening of the BME in the talus and calcaneus (Fig. 3B). Bone endocrine assessment exposed low ALP activity (17 U/L, lower limit 35 U/L) and elevated PLP (38.5 μg/L, upper limit 18.5 μg/L) as shown in Fig. 3B. TNSALP mutation analyses showed a heterozygous ALPL mutation (c.1001G>A (p.Gly334Asp)). The patient had suffered from early loss of permanent teeth at the age of 40 years, but had not previously shown any other symptoms of HPP. Subsequently, bisphosphonate therapy was stopped, and the patient was treated with teriparatide. After 3 months, ALP, BAP, and osteocalcin levels had slightly increased, whereas the PLP value had decreased (Fig. 3B). Although her phosphate level had increased to the upper reference value, her calcium and PTH levels remained in the reference range. A new MRI showed that the BME in the talus and calcaneus had completely resolved. This case suggests that patients, who seem to be asymptomatic HPP ‘‘carriers’’ without any clear signs of HPP, can develop a complication in bone healing, such as permanent BME after an ankle sprain. In this case, the bisphosphonate therapy had a rather negative effect on the clinical symptoms and the size of the BME. A similar case report in which HPP was unmasked by bisphosphonate therapy was published in 2011.^25^

## Case 2

**Figure 4:**
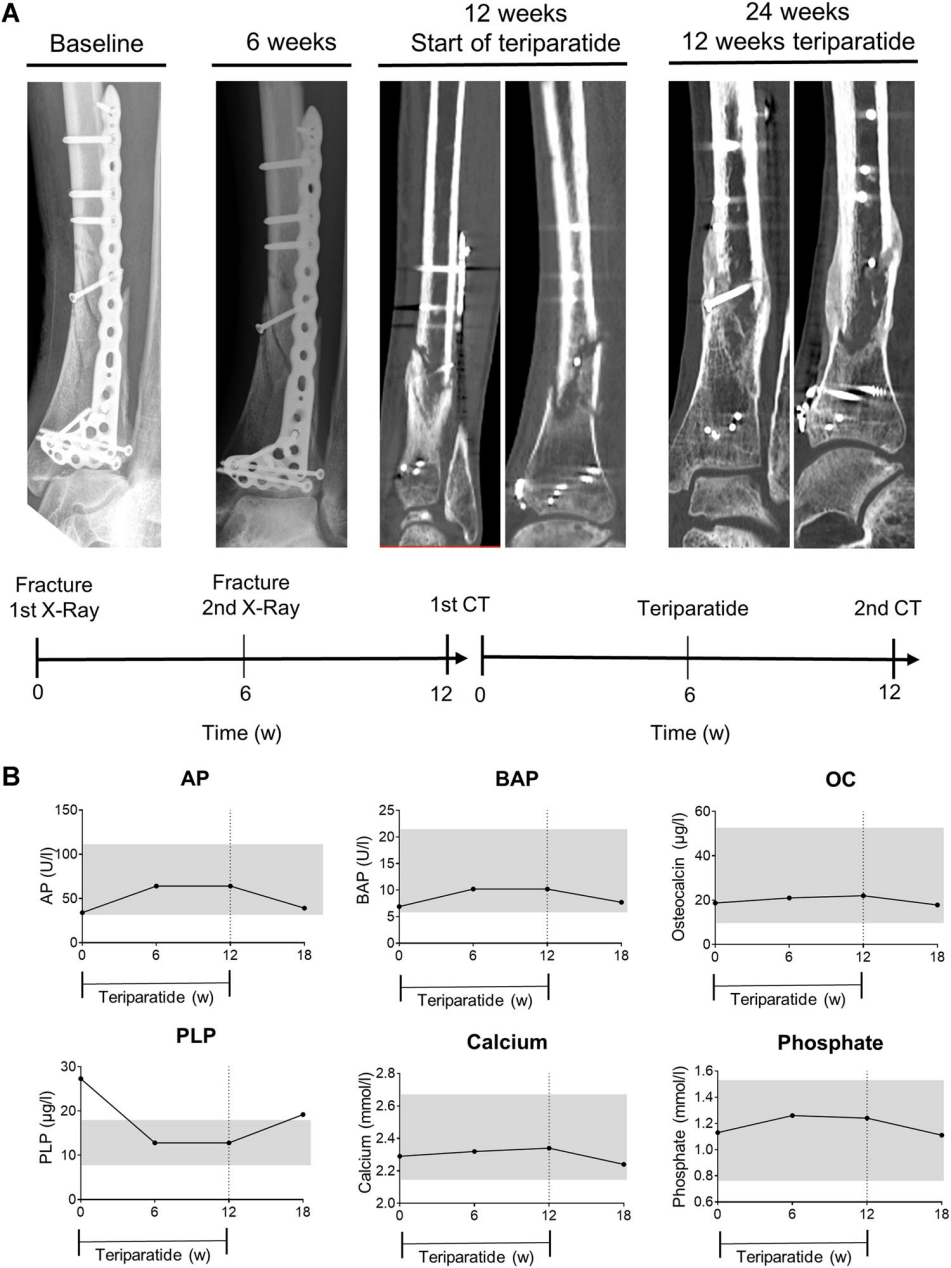
Case 2. (*A*) Radiographs and CT images of the right distal tibia postoperatively and at different time points. Treatment with teriparatide was started 3 months postoperatively. (*B*) Biochemical response with levels of alkaline phosphatase (ALP), bone‐specific alkaline phosphatase (BAP), osteocalcin (OC), pyridoxal 5’‐phosphate (PLP), calcium, and phosphate before, during, and after therapy with teriparatide. Grey bars represent reference range. The CT showed adequate bone healing under teriparatide treatment and the laboratory values displayed a significant response.

This 48‐year‐old male patient had suffered from a low‐energy fracture at the distal tibia that was treated with a plate osteosynthesis (Fig. 4A). After 6 weeks and 12 weeks, a radiograph and a CT showed no adequate bone callus formation. The patient presented at our clinic at week 12 postoperatively. Laboratory assessment revealed moderate low ALP activity (28 U/L, lower limit 40 U/L) and elevated PLP (27.3 μg/L, upper limit 18.5 μg/L) as shown in Fig. 3B. The patient did not display any other symptoms of HPP. TNSALP mutation analyses showed a heterozygous ALPL mutation (c.455G>A (p.R152H)). Because of the fracture healing complication with inadequate bone callus formation, the patient was then treated with teriparatide to accelerate fracture consolidation. After 6 weeks of therapy, the ALP level had almost doubled, osteocalcin levels had increased, and PLP levels had decreased to values within the reference range (Fig. 4B). Calcium, phosphate, and PTH remained in the reference range during the time of treatment. After 12 weeks of treatment, a CT of the fracture zone showed significant induction of bone callus formation. After cessation of therapy, ALP and PLP levels returned to baseline levels.

## Case 3

**Figure 5:**
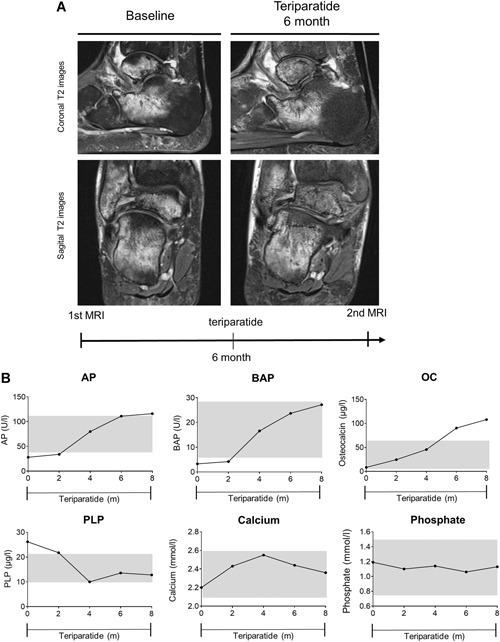
Case 3. (*A*) Coronal T2 (top) and sagittal T2 (bottom) MR images of the right foot at initial presentation and at follow‐up after 6 months. (*B*) Biochemical response with levels of alkaline phosphatase (ALP), bone‐specific alkaline phosphatase (BAP), osteocalcin (OC), pyridoxal 5’‐phosphate (PLP), calcium, and phosphate after cessation of treatment with denosumab and methotrexate and onset of therapy with teriparatide. Grey bars represent reference range. The MRI showed no clear improvement, although a significant biochemical response was observed.

The 68‐year‐old female patient had suffered from multiple stress fractures in the past including metatarsals, talus, cuboid, and calcaneus. She had been diagnosed with seropositive rheumatoid arthritis and received methotrexate (MTX) for 10 years. She presented with a persistent BME of the right distal fibula, talus, and calcaneus (Fig. 5A). At the time of initial presentation at our clinic, she had been treated with denosumab for 2 years. Although ALP levels were low (28 U/L, lower limit 35 U/L), PLP levels were slightly increased (26.2 μg/L, upper limit 18.5 μg/L). Her family history was completely unremarkable. However, TNSALP gene sequencing revealed a heterozygous mutation (c.535G>A (p.A179T)) that had been reported to be associated with HPP in the past.^26^ The patient's family medical history was unremarkable. The *Z*‐score of the lumbar spine was only slightly reduced; the *Z*‐score at the femoral neck showed strongly reduced BMD (Table 2) similar to what has been reported for pediatric HPP patients.^3^ Denosumab and MTX therapy was stopped immediately; she was treated with teriparatide instead. A strong biochemical response was observed after this treatment change. ALP and BAP activity and osteocalcin levels constantly increased within 8 months of therapy. Likewise PLP levels decreased to normal values (Fig. 5B); PTH levels decreased and stayed on a low level for the remaining time. Calcium and phosphate were in the normal range on all measurements. An MRI of the right foot was performed after 8 months of teriparatide treatment and showed persistent symptomatic BME with no clear improvement (Fig. 5A). Similar to Case 1, this case suggests that antiresorptive therapy in HPP patients or ALPL carriers can provoke complications, such as a strong reduction of bone turnover and impaired fracture healing as reported previously.^25^

## Case 4

**Figure 6:**
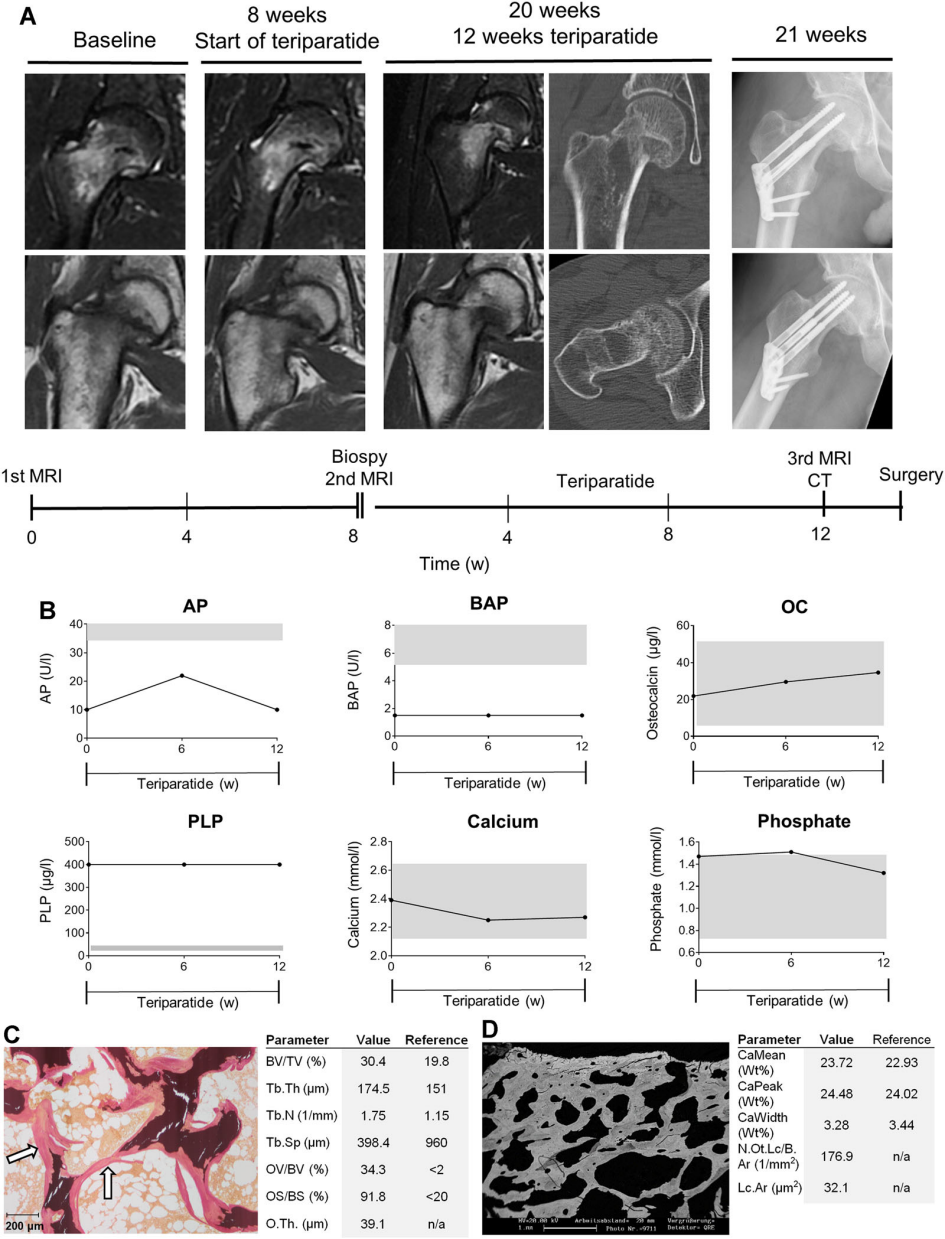
Case 4 (*A*) Coronal T2 (top) and Coronal T2 (bottom) MR images, CT images and radiographs of the right proximal femur at initial presentation, before, after 12 weeks of teriparatide and postoperatively. (B) Biochemical response with levels of alkaline phosphatase (ALP), bone‐specific alkaline phosphatase (BAP), osteocalcin (OC), pyridoxal 5’‐phosphate (PLP), calcium, and phosphate at baseline and after 12 weeks of treatment with teriparatide. Grey bars represent reference range. The teriparatide treatment failed and no clear biochemical response was detectable. (*C*) Histomorphometry images with parameters of iliac crest biopsy. Static bone histomorphometry parameters were evaluated according to the ASBMR standards. White arrows demonstrate osteoid accumulation. Reference values are according to ref.^21^ (*D*) Quantitative backscattered electron images and quantification of the overall calcium content. Reference values are according to ref.^24^

A 48‐year‐old male patient presented with a femoral neck stress fracture. The patient did not report any trauma, but stated that the pain started after a long walk. The stress fracture was treated conservatively with relief on crutches (Fig. 6A). After 8 weeks, an MRI was repeated and showed a similar BME within the fracture zone. On referral, the patient displayed ALP levels of 10 U/L (lower limit 40 U/L) and a PLP level of >400 μg/L (upper limit 18.5 μg/L) (Fig. 6B). TNSALP mutation analyses revealed two heterozygous ALPL mutations (c.746G>T (p.Gly249Val) + c.625A>T (p.Met209Leuc)). The patient showed clinical and laboratory signs of chronic kidney disease (glomerular filtration rate 43 mL/min), which had been known for 5 years and was mainly attributed to increased blood pressure. Moreover, the patient had suffered from nephrocalcinosis in the past. The patient had not been diagnosed with HPP; however, he showed many typical clinical symptoms including the loss of permanent teeth. Yet, the patient, as well as his parents and his relatives, did not report any typical HPP symptoms during childhood. No pediatric medical reports were available. On referral, the patient had not been treated with vitamin D or calcium supplementation. As recommended in the KDIGO guideline for CKD‐MBD, an iliac crest biopsy was performed to determine bone turnover and mineralization status and to rule out adynamic bone disease or other renal osteopathies. The biopsy was performed 8 weeks after onset of teriparatide therapy. Compared with reference values,^21^, ^24^ the patient showed strongly elevated osteoid volume in static bone histomorphometry (Fig. 6C), but no reduction in the calcium content (Ca mean) in quantitative backscattered electron imaging (Fig. 6D), suggesting severe osteomalacia, but no signs of adynamic bone disease. After a bone biopsy was taken, the patient was treated with low‐dose vitamin D supplementation (500 I.E. per day) and teriparatide to induce ALP activity and support bone formation. The patient also continued with relief on crutches for the next 3 months. Eight weeks after the onset of therapy ALP activity had doubled to 20 U/L (lower limit 40 U/L), while the BAP levels were unaffected. Likewise, PLP levels remained above the upper detection threshold of 400 μg/L. After 12 weeks of treatment, ALP activity was decreased to a basal level of 10 U/L (Fig. 6B). An MRI performed after 12 weeks of treatment showed no improvement, but rather a rising dislocation of the femoral neck. Additionally, CT revealed cancellous bone cavities in the femoral neck. Consequently, the patient was transferred to the trauma surgery department and subsequently treated with screw osteosynthesis (Fig. 6A).

## Discussion

In this study, we report the prevalence of fractures and BME in a large cohort of children and adults with low ALP activity and report the outcome of 4 patients with adult HPP who had been treated with teriparatide for BME or a fracture healing complication.

One limitation of the study population regards the clear distinction of “carriers” of ALPL mutation and overt HPP patients. All patients in this study showed at least one clinical symptom that has been associated with HPP in other studies.^4^, ^5^, ^27^ However, as musculoskeletal symptoms increase in the elderly population, it is somewhat problematic to attribute these symptoms completely to HPP in patients with only moderately reduced low ALP activity. Although we think that it is justified to classify the patients in this study as HPP patients, other physicians might consider some of the subjects as carriers of an ALPL mutation. For instance, we consider it reasonable to diagnose the 14‐year‐old girl with a persistent BME in the tibia diaphysis as an HPP patient because the localization is very unusual for the occurrence of a BME.^28^ However, this case also illustrates the difficulty in distinguishing ALPL mutation carriers from HPP patients.^29^ As the persistent BME was the only symptom at the time of occurrence, the patient might also be considered a mutation carrier by other physicians.

The prevalence of BME in a large cohort of patients with low ALP activity has not been reported. In our cohort, 13 of 50 patients (26%) had a history of BME that had been diagnosed with MRI. Two patients that presented with BME during the study period showed remission after 3 months without specific therapy. Interestingly, previous reports of BME in HPP have reported patients that show some, but not all signs of CRMO^9^, ^10^ with persistent BME at different localizations. However, the correct diagnosis of these inflammatory bone lesions in clinical practice can be difficult because diagnostic imaging, including MRI and bone scans, is not specific enough and may not be sufficient.^30^ Hence, a biopsy has to be undertaken and a broad microbial and laboratory workup should be performed. In this context, HPP seems to be a relevant cause or differential diagnosis for BME, particularly in younger patients. Special attention should be paid to borderline or marginally reduced ALP values that can be missed in clinical practice because the lower reference range is often not defined precisely or lowered values are overlooked.

As many adult HPP patients present with persistent musculoskeletal pain, it is possible that some of these symptoms are in fact associated with BME and may be missed on radiographs. In this context, it is important to mention that BME is increasingly identified using MRI in various clinical settings with the clinical presentation of bone pain,^31^, ^32^ including patients with inflammatory arthritis or osteoarthritis.^33^, ^34^ However, no adequate large epidemiological studies have been performed to date. Hence, it is likely that BME is, in general, underdiagnosed and underreported. However, studies suggest that most patients are 40 to 60 years of age at the onset of BME, and that the hip, knee, and feet are most commonly affected. Interestingly, it has also been reported that BME is more common in patients with osteogenesis imperfecta.^35^ Although the pathophysiology of BME is not completely understood, it has been suggested that local ischemia may initiate a high bone turnover state resulting in BME.^36^ Histological studies show increased bone resorption, but also increased osteoid indices,^37^ suggesting that a mineralization defect might be an important risk factor. In this context, reduced bone mineralization and low turnover caused by low ALP activity in HPP patients might constitute a predisposition to BME. However, no epidemiological studies have been performed to date that allow the conclusion that BME is more frequent in ALPL mutation carriers or HPP patients than in young athletes. Nonetheless, we recommend that HPP should always be taken in consideration in younger patients where BME seems to be very rare, particularly at an unusual localization such as the diaphysis.

Treatment options for BME in general are not well‐established. Often patients are treated conservatively, with an avoidance of weight‐bearing and with adequate analgesics. In general, a balanced vitamin D and calcium homeostasis has to be ensured because vitamin D deficiency seems to be an important risk factor.^38^ Antiresorptive drugs, including bisphosphonates and denosumab, have been shown to be useful in the treatment of persistent BME.^39^, ^40^, ^41^, ^42^, ^43^ However, antiresorptive medication has to be avoided in HPP because of its suppression of ALP activity. A case of atypical femoral fracture under antiresorptive therapy in an HPP patient has been published; it shows the importance of avoiding antiresorptive therapy in all carriers of an ALPL mutation or overt HPP patients.^25^ In this study, 2 patients (case 1 and case 3) had been treated with antiresorptives. Case 1 showed a deterioration of BME under therapy, and only improved after cessation of therapy and treatment with teriparatide. Case 3 had used denosumab and had also been treated with MTX for many years. MTX has been shown to inhibit osteoblastic differentiation by suppressing ALP activity.^44^ This means that the patients had received two medications that are potentially supressing ALP activity. We therefore recommend excluding HPP by measuring ALP activity and PLP in cases of borderline values in all patients that are treated with an antiresorptive drug for BME. Although the osteoanabolic drug, teriparatide was shown to reduce bone edema in one single case of a patient with BME at the hip^45^ and showed good clinical improvement in our case 1, this should be only considered in adult HPP patients with persistent BME. In pediatric patients, treatment with teriparatide is generally not recommended because the development of osteosarcoma during the rapid phase of longitudinal skeletal growth has been found in rats.^46^ In younger HPP patients, temporary AA treatment might be considered for persistent symptomatic BME as it was recently found that AA shows good effects on fracture healing in 2 adult HPP patients.^47^ Yet, there are no data available so far to draw any conclusions on the effects of teriparatide and AA on BME in HPP patients.

Fracture healing complications are a major problem in HPP patients. In our patient cohort, which mostly consisted of moderately affected adult HPP patients, 28 of 50 patients had a history of a fracture. This is consistent with the prevalence of fractures in other studies.^4^ Three of 12 patients developed a fracture healing complication in the 2‐year period of observation. We treated all of these patients with teriparatide and observed various clinical and biochemical responses. Yet, it has to be emphasized that the 3 patients were completely different in terms of secondary diseases, prior medical treatment, and severity of HPP.

Case 2 showed good healing of the distal tibia fracture with adequate formation of bony callus after receiving treatment with teriparatide. Moreover, although the serum ALP levels almost doubled for the time of treatment and reached values in the normal range, PLP levels decreased from elevated levels to normal levels. Hence, this patient benefited significantly from the temporary treatment. After stopping the therapy, ALP levels decreased and PLP levels slowly increased. A few case reports have reported treatment of adult HPP with teriparatide. The first case report was of a 56‐year‐old white woman who benefited biochemically and radiographically from a treatment period of 18 months.^17^ Another case report found a lack of sustained response in a 53‐year‐old woman with strongly decreased ALP values.^16^ Yet, it is not clear whether this could be explained by an inefficacy of the drug or by noncompliance of the patient. The most recent study also reported some benefit for 2 adult female HPP patients.^13^ Interestingly, all patients who have been treated with teriparatide have been woman.^12^, ^13^, ^14^, ^15^, ^16^, ^17^ Our study is therefore the first to report the effects of teriparatide on male adult patients. However, there is no proof of a different response in female and male patients considering the current literature.

Although case 1 and case 2 benefited clinical and biochemically from treatment, case 3 showed only a biochemical response and case 4 showed neither clinical nor clear biochemical improvement. Although case 3 used two medications that potentially supress ALP activity as discussed above, case 4 was the most severe form of adult HPP in the study cohort, with ALP activity below 10 U/L and strongly elevated PLP levels. However, because the diagnosis was established during adulthood, he was not suitable for AA therapy as there is currently no approved therapy for adult‐onset HPP in Europe. Moreover, the patient had an overlapping bone disease caused by severe HPP and CKD‐MBD. This can be one reason why the treatment with teriparatide showed no clinical response and only a moderate temporary biochemical response with an increase in ALP serum levels (10 U/L to 22 U/L). Another reason could be the compound heterozygous for TNSALP defect. In this case, the complex interaction of both mutated alleles might inhibit the effect of teriparatide as previously suggested.^16^

In summary, we show that adult patients with mild forms of HPP and low to moderately increased PLP levels can benefit from teriparatide treatment. However, the effects in patients with severe HPP and high PLP levels or with associated diseases affecting bone quality, such as rheumatoid arthritis or CKD‐MBD, seem to be limited and have to be evaluated in larger patient cohorts. Also, an evaluation of the effect of AA on BME and fracture healing in cases of compromised bone health would be an interesting subject for future studies.

## Disclosures

Florian Barvencik receives speaker and consultant fees from Alexion, Amgen, Lilly, and MSD. All other authors state that they have no conflicts of interest.

## Supporting information

Supporting information.
