# Supplementary material for: Outcome of Teriparatide Treatment on Fracture Healing Complications and Symptomatic Bone Marrow Edema in Four Adult Patients With Hypophosphatasia
Source: JBMR Plus. 2019 Aug 28;3(8):e10215. doi: 10.1002/jbm4.10215 (PMC6715780; doi:10.1002/jbm4.10215)
Supplement: Supplementary file 1 — Supporting information. [file JBM4-3-na-s001.docx]

| Case | Sex | Age at onset of symptoms | Localization | Duration  (Remission of pain) | Pain intensity (VAS) at diagnosis | Mutation  (amino acid change) | PLP (µg/l) | AP (U/L) |
| --- | --- | --- | --- | --- | --- | --- | --- | --- |
| 1  (Case 1) | f | 55 | Talus and Calcaneus | 6 month | 6 | c.1001G>A  (p.Gly334Asp) | 38,5 | 17 |
| 2  (Case 3) | f | 69 | Distal fibula, talus, and calcaneus | 12 month | 5 | c.535G>A  (p.A179T) | 26,2 | 28 |
| 3  (Case 4) | m | 49 | Femoral head / Stress fracture | Surgery after 5 month | 3 | c.746G>T & c.625A>T  (p.Gly249Val & p.Met209Leu) | 400 | 10 |
| 4 | m | 15 | Talus | 4 month | 5 | c212G>A  (p.R71H) | 61 | 84 |
| 5 | f | 56 | Femoral condyles and tibial plateau | 6 month | 4 | c.500C>T  (p.T167M) | 20,2 | 18 |
| 6 | f | 40 | Femuoral head | 6 month | 6 | c.1132>T  (p.D378Y) | 45,7 | 25 |
| 7 | f | 58 | Distal tibia | 3 month | 8 | c.571G>A  (c.1171delC) | 77,5 | 10 |
| 8 | f | 32 | Metatarsale IV | 3 month | 7 | c.199C>T  (p.Ala40Val/p.A40V) | 889 | 10 |
| 9 | f | 27 | Talus and Calcaneus | 6 month | 4 | c.340G>A  (p.A114T) | 105 | 26 |
| 10 | f | 58 | Tibia plateau | 5 month | 5 | c.571G>A  (p.E191K) | 27,2 | 28 |
| 11 | f | 54 | Talus and Calcaneus | 7 month | 4 | C.571G>A  (p.E191K) | 184,8 | 18 |
| 12 | f | 45 | Distal Femur | 4 month | 5 | c.3G>A  (p.Met1) | 32,2 | 22 |
| 13 | f | 15 | Tibia Diaphysis | 9 month | 5 | c.746G>T  (p.Gly249Ser) | 59,7 | 58 |
| 14 | m | 59 | Tibia plateau | 7 month | 4 | c.571G>A  (p.E191K) | 23,6 | 31 |

**Supplemental Table 1:** Characteristics of patients with BME
